# The emergence of novel sparrow deltacoronaviruses in the United States more closely related to porcine deltacoronaviruses than sparrow deltacoronavirus HKU17

**DOI:** 10.1038/s41426-018-0108-z

**Published:** 2018-06-06

**Authors:** Qi Chen, Leyi Wang, Chenghuai Yang, Ying Zheng, Phillip C. Gauger, Tavis Anderson, Karen M. Harmon, Jianqiang Zhang, Kyoung-Jin Yoon, Rodger G. Main, Ganwu Li

**Affiliations:** 1 Department of Veterinary Diagnostic and Production Animal Medicine, College of Veterinary Medicine, Iowa State University, Ames, IA 50010 USA; 2 Department of Veterinary Clinical Medicine and the Veterinary Diagnostic Laboratory, College of Veterinary Medicine, University of Illinois, Urbana, IL 61802 USA; 3 National Animal Disease Center, USDA-ARS, Ames, IA 50010 USA; 4 State Key Laboratory of Veterinary Biotechnology, Harbin Veterinary Research Institute, Chinese Academy of Agricultural Sciences, 678 Haping Street, Harbin, 150069 China

Dear Editor,

Coronaviruses (CoVs) are single-stranded, positive-sense, enveloped RNA viruses belonging to family *Coronaviridae* and subfamily *Coronavirinae*, which consist of four genera: *Alphacoronavirus*, *Betacoronavirus*, *Gammacoronavirus*, and *Deltacoronavirus*^1^. Each genus of CoVs has been found in diverse animal species and causes different types of disease, including respiratory, enteric, hepatic, renal, reproductive and neurological diseases^2^. To date, all known CoVs detected from birds belong to genus *Gammacoronavirus* or *Deltacoronavirus*, supporting the previously proposed model of CoV evolution in which avian CoVs serve as the gene source for *Gammacoronavirus* and *Deltacoronavirus*^3^.

The genus *Deltacoronavirus* consists of different mammalian (Asian leopard cat CoV, Chinese ferret badger CoV, and porcine CoV HKU15) and avian (bulbul CoV HKU11, thrush CoV HKU12, munia CoV HKU13, white-eye CoV HKU16, sparrow CoV HKU17, magpie-robin CoV HKU18, night heron CoV HKU19, wigeon CoV HKU20, and common moorhen CoV HKU21) CoVs^3^. Initially detected in a surveillance study, porcine CoV HKU15, now commonly referred to as porcine deltacoronavirus (PDCoV), has been identified as one of the major enteric pathogens causing diarrhea in pigs since 2014^4, 5^. Both clinical and experimental evidence demonstrated that PDCoV is pathogenic to swine and causes similar lesions in the pig intestine as porcine epidemic diarrhea virus (PEDV)^6, 7^. PDCoV strains reported from the United States and other countries had 97.1–99.9% nucleotide identity at the whole-genome level^8^, suggesting that a single genotype is circulating worldwide. Regarding sparrow deltacoronavirus (SpDCoV), one sparrow CoV strain, HKU17-6124, has been reported thus far^3^. The genomic diversity of sparrow deltacoronaviruses remains unclear. We report the detection and genetic characterization of novel SpDCoV in fecal samples collected from sparrows found on swine farms in the midwestern United States.

In January 2017, multiple batches of porcine oral fluid (pen-based) and floor-picked fecal samples were submitted from a grow-finish stage pig farm in Illinois for routine health monitoring. Unexpectedly, multiple oral fluid samples and one fecal sample were tested positive for PDCoV (the Ct value ranged between 34 and 39) by duplex PEDV and PDCoV real-time RT-PCR (see Technical Appendix). However, no clinical diarrhea or other signs were observed among pigs on the index farm, which prompted us to further investigate the source of positive PCR results. Since the pigs were housed in hoop-style buildings with uncontrolled access for wild bird entry and congregation, fecal samples were also collected from 10 sparrows found inside the pig barn (ISU690-1 to −10) and were submitted to our laboratory for testing in January 2017, along with 8 feed samples (ISU690-11 to −18). Surprisingly, 9/10 fecal samples were positive using PCR targeting the PDCoV N gene, with a Ct of 26.3–37.3, while all 8 feed samples were negative. Four additional sparrow fecal samples (ISU42824-1 to −4) collected in June 2017 from the same farm were tested, and one of the samples (ISU42824-3) was positive by PDCoV N gene-based PCR, with a Ct of 23.6.

Similarly, in an unrelated grow-finish swine farm in Minnesota, two oral fluid samples collected from clinically healthy pigs at the end of October 2017 were tested positive (Ct of 33.3 and 34.4), and one sparrow fecal sample (ISU73347-2) collected in early November 2017 from that barn were tested positive (Ct of 22.6) using a PDCoV N gene-based PCR.

The PDCoV PCR-positive sparrow samples then underwent next-generation sequencing (NGS) using an Illumina MiSeq platform (see Supplementary methods). After using an in-house bioinformatics analysis pipeline (Supplementary methods), four nearly complete genomic sequences of SpDCoV (ISU690-4, ISU690-7, ISU42824, and ISU73347; GenBank accession numbers MG812375, MG812376, MG812377, and MG812378, respectively) were obtained. The genome organizations of all four SpDCoV strains are similar to those of sparrow CoV HKU17 and PDCoV with the characteristic gene order 5′-replicase ORF1ab, spike (S), envelope (E), membrane (M), and nucleocapsid (N)-3′ (Fig. 1a). Similar to HKU17, the four SpDCoV strains contain three ORFs downstream of N encoding three nonstructural proteins (NS7a, NS7b, and NS7c), as illustrated in Fig. 1a.

**Fig. 1:**
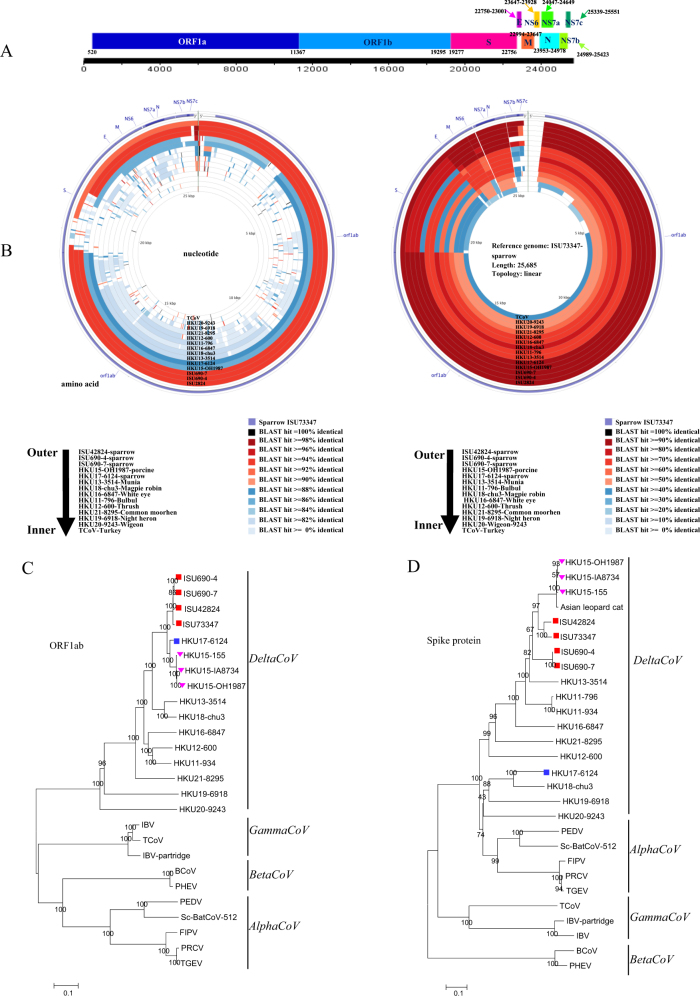
Characterization of sparrow deltacoronavirus (SpDCoV). **a** Genome organization of SpDCoV ISU73347. **b** Nucleotide and amino acid comparison of ISU73347 with three sparrow coronaviruses and other reference strains (PDCoV strain HKU15-OH1987, KJ462462; sparrow CoV strain HKU17-6124, NC_016992; munia CoV strain HKU13-3514, NC_011550; magpie robin CoV strain HKU18-chu3, NC_016993; white-eye CoV HKU16-6847, JQ065044; bulbul CoV strain HKU11-796, FJ376620; thrush CoV strain HKU12-600, NC_011549; common moorhen CoV strain HKU21-8295, NC_016996; night heron CoV strain HKU19-6918, NC_016994; wigeon CoV strain HKU20-9243, JQ065048; TCoV, turkey coronavirus, NC_010800). Analysis was completed using CGView Comparison Tool software. The corresponding strains/samples for rings are detailed on the bottom. ORF open reading frame, S spike, E envelope, M membrane, N nucleocapsid, NS6, NS7a, NS7b, and NS7c represent nonstructural proteins 6, 7a, 7b, and 7c, respectively. **c** Phylogeny of ORF1ab amino-acid sequences. **d** Phylogeny of spike protein sequences. The sparrow deltacoronavirus strains identified in this study are indicated with red squares, HKU17-6124 is marked with a blue square, and three HKU15 strains are marked with a pink triangle. The phylogeny was inferred using the neighbor-joining method in MEGA version 7.0. Statistical support was obtained using bootstrap resampling (1000 replications) and is drawn on the inferred tree. Reference sequences representing coronavirus diversity were obtained from NCBI GenBank (PEDV, porcine epidemic diarrhea virus, NC_003436; Sc-BatCoV-512, *Scotophilus* bat coronavirus 512, NC_009657, TGEV transmissible gastroenteritis virus, AJ271965, FIPV feline infectious peritonitis virus, NC_002306, PRCV porcine respiratory coronavirus, DQ811787, PHEV porcine hemagglutinating encephalomyelitis virus, DQ011855, BCoV bovine coronavirus, NC_003045, IBV infectious bronchitis virus, NC_001451, IBV-partridge partridge coronavirus, AY646283, TCoV turkey coronavirus, NC_010800; bulbul, CoV strain HKU11-796, FJ376620; bulbul CoV strain HKU11-934, FJ376619; thrush CoV strain HKU12-600, NC_011549; munia CoV strain HKU13-3514, NC_011550; PDCoV strain HKU15-OH1987, KJ462462; PDCoV strain HKU15-IA8734, KJ567050; PDCoV strain HKU15-155, JQ065043; white-eye CoV strain HKU16-6847, JQ065044; sparrow CoV strain HKU17-6124, NC_016992; magpie robin CoV strain HKU18-chu3, NC_016993; night heron CoV strain HKU19-6918, NC_016994; wigeon CoV strain HKU20-9243, JQ065048; common moorhen CoV strain HKU21-8295, NC_016996). The scale bar represents 0.1 amino acid substitutions per site

Whole-genome nucleotide sequence comparison showed that SpDCoV ISU73347 shares a higher identity with ISU42824 (93.9%) and ISU690-4 and −7 (92.5%) than with PDCoV (83.9%) and HKU17 (82.1%), and it has an even lower identity with other avian deltacoronaviruses, such as HKU13 (70.7%), HKU16 (69.1%), and HKU18 (69.0%) (Fig. 1b, Supplementary Table S1). SpDCoV ISU690-4 and ISU690-7 share 99.8% whole-genome nucleotide identity with each other, whereas both strains share 93.1%, 92.5%, 83.2%, and 82.7% identity with ISU42824, ISU73347, PDCoV, and HKU17, respectively, and they share an even lower identity with other avian deltacoronaviruses, such as HKU13 (71.4%), HKU16 (69.3%), and HKU18 (69.4%). These data suggest that the newly identified SpDCoV ISU690-4/ISU690-7, ISU42824, and ISU73347 represent novel SpDCoV strains. To exclude the possible issue of contamination of sparrow fecal samples with porcine samples, all fecal samples directly collected from swine were tested and were negative for both SDCoV and SpDCoV. In addition, Sanger sequencing of four RT-PCR products of the ORF1ab, M, and N genes of SpDCoV showed that the sequences had 100% identity to those from NGS.

The four SpDCoV strains identified in this study share a slightly higher identity (94.4–94.6% and 96.0–96.4%) to a sparrow CoV strain HKU17-6124 than to PDCoV strains (93.4–93.6% and 95.3–95.8%) in their ORF1ab and N proteins, respectively (Fig. 1b and Supplementary Table S2). In contrast, the four SpDCoV strains share a significantly lower identity to sparrow CoV HKU17 (58.2–58.6%) than to three PDCoV strains (82.7–87.7%) in their S protein (Fig. 1b and Supplementary Table S2). In addition, they share a relatively lower identity (90.7–91.5% and 93.5–95.5%) to sparrow CoV HKU17 than the PDCoV strains (94.9–95.8% and 96.1–98.1%) in their E and M proteins, respectively (Supplementary Table S2). These findings were also confirmed by phylogenetic tree analysis of amino-acid sequences in which the four SpDCoV strains clustered together and were closely related to PDCoV and sparrow CoV HKU17 strains in ORF1ab, E, M, and N trees (Fig. 1c and Supplementary Figure S1). However, the four SpDCoV strains were close to PDCoV but distant from sparrow CoV HKU17 in the S tree (Fig. 1d). In the case of non-structural proteins (NS6, NS7a, and NS7b), the SpDCoV strains share a high amino-acid identity with each other. However, SpDCoV ISU690-4/7 has 76.2% and 79% identity to ISU73347 and ISU42824, respectively, in NS7c, which is much lower than the 97.2% identity shared between ISU73347 and ISU42824 (Supplementary Table S3). An 18-nt deletion in the NS7c gene of ISU690-4 and -7 resulted in a protein that was 6 amino acids shorter than that of ISU73347 and ISU42824 in length (Supplementary Figure S2). Overall, these data further support that the sparrow CoVs identified in our study are novel SpDCoV.

Recombination analysis revealed that PDCoV and sparrow CoV HKU17 are highly variable from SpDCoVs in two regions. The most prominent variable region is located in the S-E-M gene region, and another region is located within the ORF1ab gene (~2675 nt to 3435 nt) (Supplementary Figure S3). However, there is no obvious pattern showing that the emergence of SpDCoV strains identified in the study was through recombination between PDCoV and sparrow CoV HKU17.

Comparison of PDCoV PCR primer and probe sequences (targeting the N gene) with SpDCoV sequences revealed that the probe sequence was exactly identical to the sequence observed in SpDCoV, and only 2 and 0/1 nucleotide mismatches were observed in the forward and reverse primers, respectively (Supplementary Figure S4). Therefore, PDCoV real-time RT-PCR can cross-react with SpDCoV, which complicates the molecular detection of PDCoV. It is plausible that contamination with sparrow feces containing SpDCoVs caused non-specific positive results for PDCoV when the oral fluid and fecal samples collected from clinical healthy pigs were tested using PEDV/PDCoV duplex RT-PCR. This also raises a concern regarding the specificity of detecting PDCoV from environmental and environmentally contaminated samples. To avoid the cross-reaction issue, further modification of the primers and probe for PDCoV real-time RT-PCR is needed.

PDCoV, as a causative agent for pig diarrhea, has been reported in many countries, including the United States, Canada, Japan, South Korea, Thailand, Vietnam, Mainland China, and Laos, indicating that PDCoV is widespread in pig populations. However, all known PDCoVs to date have been relatively conserved and shared 97.1–99.9% nucleotide identity at the whole genome level. In contrast, the SpDCoV strains identified in the present study (ISU690-4/7, ISU42824, and ISU73347) as well as one previously identified sparrow CoV strain, HKU17-6124, exhibited higher genetic diversity (82.1–93.9% nucleotide identity at the whole-genome level). Further studies to understand the genetic diversity of SpDCoV by sequencing more SpDCoV strains are warranted. Another important finding is that, due to the relative conservation between PDCoV and SpDCoV N gene sequences in some regions, the PDCoV N gene-based PCR used in this study can cross-react with SpDCoV. Thus, PDCoV PCR results should be interpreted carefully when pig samples that can be contaminated by bird feces or other excretions are tested. The SpDCoV sequence data reported in this study may help determine the evolutionary relationship of various DCoVs and help to understand the interspecies transmission of DCoVs in future studies.

## Electronic supplementary material

Supplemental Methods, Tables and Figures
