# Supplementary material for: The emergence of novel sparrow deltacoronaviruses in the United States more closely related to porcine deltacoronaviruses than sparrow deltacoronavirus HKU17
Source: Emerg Microbes Infect. 2018 Jun 6;7:105. doi: 10.1038/s41426-018-0108-z (PMC5988828; doi:10.1038/s41426-018-0108-z)
Supplement: Supplementary file 1 — Supplemental Methods, Tables and Figures [file 41426_2018_108_MOESM1_ESM.docx]

**Supplementary Methods**

**DNA/RNA purification and PEDV-PDCoV PCR**

Total DNA/RNA from fecal or feed samples was extracted as previously described ^1^, and the extracted DNA/RNA was eluted into 50ul elution buffer. PEDV-PDCoV PCR developed and routinely performed at the ISU VDL is designed to simultaneously detect and differentiate both PEDV and PDCoV in a sample with 2 pairs of primers and probe specifically targeting each virus, respectively. Relatively conserved regions of nucleocapsid (N) gene of PEDV and N gene of PDCoV were used to develop primers and probes. The primers and probes (sequences in parentheses) are: PDCoV forward primer (5’-CCTACTACTGACGCGTCTTGGTT-3’), PDCoV reverse primer (5’-TGCCACGAAACTGAGGATGA-3’), PDCoV probe labeled with VIC reporter dye (5’/56-VIC/TGCTCAAAGCTCAAAAC/MGB/-3’), PEDV forward primer (5’-CGCAAAGACTGAACCCACTAACCT-3’), PEDV reverse primer (5’-TTGCCTCTGTTGTTACTTGGAGAT-3’), PEDV probe labeled with FAM reporter dye (5’-/56-FAM/TGTTGCCAT/ZEN/TACCACGACTCCTGC/3IABKFQ-3’). All collected samples were subject to PEDV-PDCoV PCR. PCR reaction comprises 20ul volume with Taqman^®^ Fast Virus 1-step Master Mix (Thermo Fisher Scientific, Waltham, MA) reagents.

**NGS**

To further characterize the RNA viruses detected by the PDCoV PCR portion of PEDV-PDCoV PCR, bird fecal samples that tested PDCoV positive in PCR were subject to next generation sequencing. Specifically, DNA in the extracted DNA/RNA from these samples was removed with RNase-Free DNase Set (Qiagen, Valencia, CA), and reagent residual was then removed from the remaining RNA with Agencourt^®^RNAClean^®^ XP (Beckman Coulter, Indianapolis, IN) kit according to the kit manual. The library was prepared with NEXTflex™ Rapid RNA-Seq Kit (Bioo Scientific, Austin, TX) until “step D” according to kit manual with minor modification, and followed with Nextera XT DNA library preparation kit (Illumina, San Diego, CA). Normalized library was sequenced on MiSeq platform (Illumina, San Diego, CA) with 300-cycle MiSeq Reagent Micro Kit V2 (Illumina, San Diego, CA).

**Bioinformatics analysis**

Raw sequencing data were subject to data cleaning by removing adapters, trimming low quality ends, depleting sequences with length < 36nt, and sequencing quality analysis with FastQC ^2^. Taxonomy of cleaned reads were classified using Kraken v0.10.5-beta ^3^. Reads of particular/interested viruses were extracted from the kraken classification results as candidate reads of that taxon. Particularly, DCoV fragments were extracted and were *de novo* assembled with SPAdes (v 3.5.0) based on candidate reads ^4^.

**Results**

Further comparison of deduced amino acid sequences showed that ORF1ab, S, E, M and N proteins of these novel SpDCoV strains share 99.0%-99.3%, 86.1%-93.4%, 98.3%-99.2%, 96.8%-98.7%, and 99.6% identity with each other, respectively (Supplementary Table S2). ISU690-4 and ISU690-7 share 100% identity in all structural and non-structural proteins. ISU73347 and ISU42824 are more closely correlated to each other than to ISU690-4/7 in their S protein, while ISU73347 and ISU690-4/7 share a higher identity (98.7%) to each other than to ISU42824 (96.8%) in their M protein (Supplementary Table S2). Among the different proteins, the N protein is highly conserved among all four SpDCoV strains.

**References**

1 Chen, Q., Li, G., Stasko, J. *et al.* Isolation and characterization of porcine epidemic diarrhea viruses associated with the 2013 disease outbreak among swine in the United States. *J Clin Microbiol* **52**, 234-243, doi:10.1128/JCM.02820-13 (2014).

2 Bolger, A. M., Lohse, M. & Usadel, B. Trimmomatic: a flexible trimmer for Illumina sequence data. *Bioinformatics* **30**, 2114-2120, doi:10.1093/bioinformatics/btu170 (2014).

3 Wood, D. E. & Salzberg, S. L. Kraken: ultrafast metagenomic sequence classification using exact alignments. *Genome Biol* **15**, doi:ARTN R46

10.1186/gb-2014-15-3-r46 (2014).

4 Bankevich, A., Nurk, S., Antipov, D. *et al.* SPAdes: a new genome assembly algorithm and its applications to single-cell sequencing. *J Comput Biol* **19**, 455-477, doi:10.1089/cmb.2012.0021 (2012).

**Supplementary Table S1**. Comparison of nucleotide identity of complete genome*

|  | ISU73347 | ISU690-4 | ISU690-7 | ISU42824 |
| --- | --- | --- | --- | --- |
| ISU73347 | - | 92.5 | 92.4 | 93.9 |
| ISU690-4 | 92.5 | - | 99.8 | 93.1 |
| ISU690-7 | 92.4 | 99.8 | - | 93.1 |
| ISU42824 | 93.9 | 93.1 | 93.1 | - |
| HKU15-OH1987 | 83.9 | 83.2 | 83.1 | 83.7 |
| HKU17-6124 | 82.1 | 82.7 | 82.7 | 82.3 |
| HKU13-3514 | 70.7 | 71.4 | 71.4 | 71.0 |
| HKU16-6847 | 69.1 | 69.3 | 69.3 | 69.0 |
| HKU18-chu3 | 69.0 | 69.4 | 69.4 | 69.2 |
|  |  |  |  |  |

*Comparison of pairwise nucleotide identity (%) was completed using a MegAlign ClustalW method.

SpDCoV strains ISU73347, ISU690-4, ISU690-7, and ISU42824; PDCoV strain HKU15-OH1987 (GenBank accession # KJ462462); sparrow CoV strain HKU17-6124 (NC_016992); munia CoV strain HKU13-3514 (NC_011550); white-eye CoV strain HKU16-6847 (JQ065044); and magpie robin CoV strain HKU18-chu3 (NC_016993).

**Supplementary Table S2**. Comparison of amino acid identity of structural proteins*

|  | **ISU73347** | | | | |  | | **ISU690-4/7** | | | | |  | **ISU42824** | | | | |
| --- | --- | --- | --- | --- | --- | --- | --- | --- | --- | --- | --- | --- | --- | --- | --- | --- | --- | --- |
|  | ORF1ab | S | E | M | N |  | ORF1ab | | S | E | M | N |  | ORF1ab | S | E | M | N |
| **ISU73347** | - | - | - | - | - |  | | 99.0 | 86.8 | 99.2 | 98.7 | 99.6 |  | 99.0 | 93.4 | 99.2 | 96.8 | 99.6 |
| **ISU690-4/7** | 99.0 | 86.8 | 99.2 | 98.7 | 99.6 |  | | - | - | - | - | - |  | 99.3 | 86.1 | 98.3 | 96.8 | 99.6 |
| **ISU42824** | 99.0 | 93.4 | 99.2 | 96.8 | 99.6 |  | | 99.3 | 86.1 | 98.3 | 96.8 | 99.6 |  | - | - | - | - | - |
| **HKU17-6124** | 94.4 | 58.6 | 91.5 | 94.8 | 96.0 |  | | 94.6 | 58.2 | 90.7 | 95.5 | 96.4 |  | 94.5 | 58.4 | 91.5 | 93.5 | 96.0 |
| **HKU15-155** | 93.4 | 86.7 | 95.8 | 96.4 | 95.4 |  | | 93.6 | 82.7 | 94.9 | 97.7 | 95.8 |  | 93.4 | 87.6 | 94.9 | 96.1 | 95.8 |
| **HKU15-IA8734** | 93.4 | 87.0 | 95.8 | 96.8 | 95.3 |  | | 93.5 | 82.8 | 94.9 | 98.1 | 95.6 |  | 93.4 | 87.7 | 94.9 | 96.4 | 95.6 |
| **HKU15-OH1987** | 93.4 | 87.0 | 95.8 | 96.8 | 95.3 |  | | 93.5 | 82.8 | 94.9 | 98.1 | 95.6 |  | 93.4 | 87.7 | 94.9 | 96.4 | 95.6 |
| **HKU11-934** | 80.0 | 79.4 | 82.2 | 78.6 | 83.2 |  | | 80.1 | 78.7 | 81.4 | 79.0 | 83.4 |  | 79.9 | 79.5 | 82.2 | 79.0 | 83.2 |
| **HKU12-600** | 79.7 | 62.0 | 85.6 | 83.5 | 86.7 |  | | 79.7 | 61.5 | 84.7 | 83.2 | 87.0 |  | 79.6 | 61.5 | 85.6 | 84.5 | 86.9 |
| **HKU13-3514** | 82.8 | 81.4 | 78.8 | 80.9 | 83.4 |  | | 82.8 | 82.9 | 78.0 | 81.6 | 83.8 |  | 82.8 | 81.9 | 78.8 | 82.8 | 83.4 |
| **HKU16-6847** | 78.4 | 73.1 | 83.1 | 84.5 | 84.3 |  | | 78.3 | 72.2 | 82.2 | 84.8 | 84.7 |  | 78.3 | 72.8 | 83.1 | 85.8 | 84.5 |
| **HKU18-chu3** | 83.8 | 58.5 | 82.2 | 80.6 | 85.6 |  | | 83.9 | 58.3 | 81.4 | 80.9 | 86.0 |  | 83.8 | 58.7 | 82.2 | 81.2 | 85.6 |
| **HKU19-6918** | 59.2 | 53.9 | 53.4 | 66.7 | 67.0 |  | | 59.2 | 54.7 | 53.4 | 67.0 | 67.2 |  | 59.2 | 54.8 | 53.4 | 67.3 | 66.8 |
| **HKU20-9243** | 58.7 | 57.5 | 50.0 | 67.6 | 67.8 |  | | 58.7 | 57.0 | 50.0 | 67.6 | 67.9 |  | 58.7 | 57.9 | 50.0 | 67.6 | 67.6 |
| **HKU21-8295** | 74.4 | 64.1 | 72.9 | 58.3 | 74.5 |  | | 74.5 | 64.3 | 72.0 | 57.9 | 74.7 |  | 74.4 | 64.0 | 72.9 | 58.3 | 74.5 |
| **TCoV** | 40.1 | 42.3 | 33.9 | 42.7 | 42.6 |  | | 40.0 | 42.8 | 33.9 | 42.7 | 42.4 |  | 40.1 | 42.3 | 33.9 | 42.1 | 42.6 |
| **IBV** | 40.2 | 42.3 | 26.3 | 42.4 | 42.2 |  | | 40.2 | 42.0 | 26.3 | 42.4 | 42.0 |  | 40.2 | 42.2 | 26.3 | 42.1 | 42.2 |
| **IBV-partridge** | 39.8 | 42.9 | 26.3 | 43.0 | 43.0 |  | | 39.8 | 42.8 | 26.3 | 43.0 | 42.8 |  | 39.9 | 42.6 | 26.3 | 42.7 | 43.0 |
| **BCoV** | 29.9 | 32.1 | 33.1 | 41.1 | 30.2 |  | | 29.8 | 32.0 | 33.1 | 41.1 | 30.2 |  | 29.8 | 32.3 | 33.1 | 41.4 | 30.4 |
| **PHEV** | 29.8 | 34.1 | 33.1 | 40.5 | 30.2 |  | | 29.7 | 34.0 | 33.1 | 40.5 | 30.2 |  | 29.7 | 33.7 | 33.1 | 40.8 | 30.4 |
| **PEDV** | 31.8 | 43.1 | 35.6 | 40.8 | 28.4 |  | | 31.8 | 42.9 | 35.6 | 40.8 | 28.2 |  | 31.8 | 43.0 | 35.6 | 40.8 | 28.4 |
| **TGEV** | 34.2 | 40.6 | 32.2 | 29.1 | 36.6 |  | | 34.2 | 40.1 | 32.2 | 29.1 | 36.4 |  | 34.2 | 40.6 | 32.2 | 29.4 | 36.6 |
| **PRCV** | 34.0 | 51.8 | 32.2 | 29.4 | 36.6 |  | | 34.0 | 51.4 | 32.2 | 29.4 | 36.4 |  | 34.0 | 51.9 | 32.2 | 29.8 | 36.6 |
| **Sc-BatCoV-512** | 31.7 | 44.8 | 39.0 | 42.1 | 36.1 |  | | 31.7 | 43.9 | 39.0 | 42.1 | 35.9 |  | 31.8 | 44.2 | 39.0 | 41.4 | 36.1 |
| **FIPV** | 34.2 | 40.5 | 32.2 | 29.1 | 36.4 |  | | 34.2 | 40.2 | 32.2 | 29.1 | 36.2 |  | 34.2 | 40.5 | 32.2 | 29.8 | 36.4 |

*Comparison of pairwise amino acid identity (%) was completed using a MegAlign ClustalW method.

PEDV, porcine epidemic diarrhea virus, NC_003436; Sc-BatCoV-512, *Scotophilus* bat coronavirus 512, NC_009657; TGEV, transmissible gastroenteritis virus, AJ271965; FIPV, feline infectious peritonitis virus, NC_002306; PRCV, porcine respiratory coronavirus, DQ811787; PHEV, porcine hemagglutinating encephalomyelitis virus, DQ011855; BCoV, bovine coronavirus, NC_003045; IBV, infectious bronchitis virus, NC_001451; IBV-partridge, partridge coronavirus, AY646283; TCoV, turkey coronavirus, NC_010800; bulbul CoV strain HKU11-934, FJ376619; thrush CoV strain HKU12-600, NC_011549; munia CoV strain HKU13-3514, NC_011550; PDCoV strain HKU15-OH1987, KJ462462; PDCoV strain HKU15-IA8734, KJ567050; PDCoV strain HKU15-155, JQ065043; white-eye CoV strain HKU16-6847, JQ065044; sparrow CoV strain HKU17-6124, NC_016992; magpie robin CoV strain HKU18-chu3, NC_016993; night heron CoV strain HKU19-6918, NC_016994; wigeon CoV strain HKU20-9243, JQ065048; common moorhen CoV strain HKU21-8295, NC_016996.

**Supplementary Table S3**. Comparison of amino acid identity of non-structural proteins*

|  | **ISU73347** | | | | **ISU690-4/7** | | | | **ISU42824** | | | |  |
| --- | --- | --- | --- | --- | --- | --- | --- | --- | --- | --- | --- | --- | --- |
|  | NS6 | NS7a | NS7b | NS7c | NS6 | NS7a | NS7b | NS7c | NS6 | NS7a | NS7b | NS7c |  |
| **ISU73347** | | - | - | - | - | 99.1 | 95.5 | 94.6 | 76.2 | 97.2 | 96.9 | 96.6 | 97.2 |
| **ISU690-4/7** | | 99.1 | 95.5 | 94.6 | 76.2 | - | - | - | - | 98.1 | 97.3 | 98.0 | 79.0 |
| **ISU42824** | | 97.2 | 96.9 | 96.6 | 97.2 | 98.1 | 97.3 | 98.0 | 79.0 | - | - | - | - |
| **HKU17** | | 93.5 | 89.3 | 81.9 | 88.8 | 93.5 | 90.6 | 81.2 | 79.7 | 91.7 | 92.0 | 81.9 | 90.2 |
| **HKU15-155** | | 87.0 | 87.5 | NA | NA | 88.0 | 87.1 | NA | NA | 86.1 | 87.5 | NA | NA |
| **HKU15-IA8734** | | 87.0 | 85.7 | NA | NA | 88.0 | 85.7 | NA | NA | 86.1 | 86.2 | NA | NA |
| **HKU15-OH1987** | | 87.0 | 86.2 | NA | NA | 88.0 | 86.2 | NA | NA | 86.1 | 86.6 | NA | NA |
| **HKU11-934** | | 70.4 | 18.8 | 13.4 | 53.1 | 70.4 | 19.2 | 13.4 | 50.3 | 69.4 | 18.8 | 13.4 | 51.0 |
| **HKU12-600** | | 75.9 | 18.3 | 12.1 | 48.3 | 75.9 | 18.3 | 12.1 | 45.5 | 74.1 | 18.3 | 12.1 | 48.3 |
| **HKU13-3514** | | 55.6 | 18.3 | 12.8 | 53.1 | 55.6 | 18.3 | 12.8 | 53.8 | 54.6 | 18.3 | 12.8 | 54.5 |
| **HKU16-6847** | | 77.8 | 49.6 | 8.7 | NA | 77.8 | 49.6 | 8.7 | NA | 77.8 | 50.0 | 8.7 | NA |
| **HKU18-chu3** | | 69.4 | 11.6 | 29.5 | 44.8 | 69.4 | 11.6 | 28.9 | 46.9 | 70.4 | 11.6 | 29.5 | 46.2 |
| **HKU19-6918** | | 39.8 | 13.4 | 6.7 | NA | 38.9 | 13.4 | 6.7 | NA | 39.8 | 13.4 | 6.7 | NA |
| **HKU20-9243** | | 46.3 | 13.4 | 8.7 | 44.8 | 46.3 | 13.8 | 8.7 | 44.1 | 46.3 | 13.8 | 8.7 | 45.5 |
| **HKU21-8295** | | 37.0 | 16.1 | 6.7 | 9.1 | 37.0 | 16.1 | 6.7 | 9.8 | 37.0 | 15.6 | 6.7 | 8.4 |

*Comparison of pairwise amino acid identity (%) was completed using a MegAlign ClustalW method. NS6, NS7a, NS7b, and NS7c represent nonstructural protein 6, 7a, 7b, and 7c, respectively.

bulbul CoV strain HKU11-934, FJ376619; thrush CoV strain HKU12-600, NC_011549; munia CoV strain HKU13-3514, NC_011550; PDCoV strain HKU15-OH1987, KJ462462; PDCoV strain HKU15-IA8734, KJ567050; PDCoV strain HKU15-155, JQ065043; white-eye CoV strain HKU16-6847, JQ065044; sparrow CoV strain HKU17-6124, NC_016992; magpie robin CoV strain HKU18-chu3, NC_016993; night heron CoV strain HKU19-6918, NC_016994; wigeon CoV strain HKU20-9243, JQ065048; common moorhen CoV strain HKU21-8295, NC_016996.

NA: not applicable.


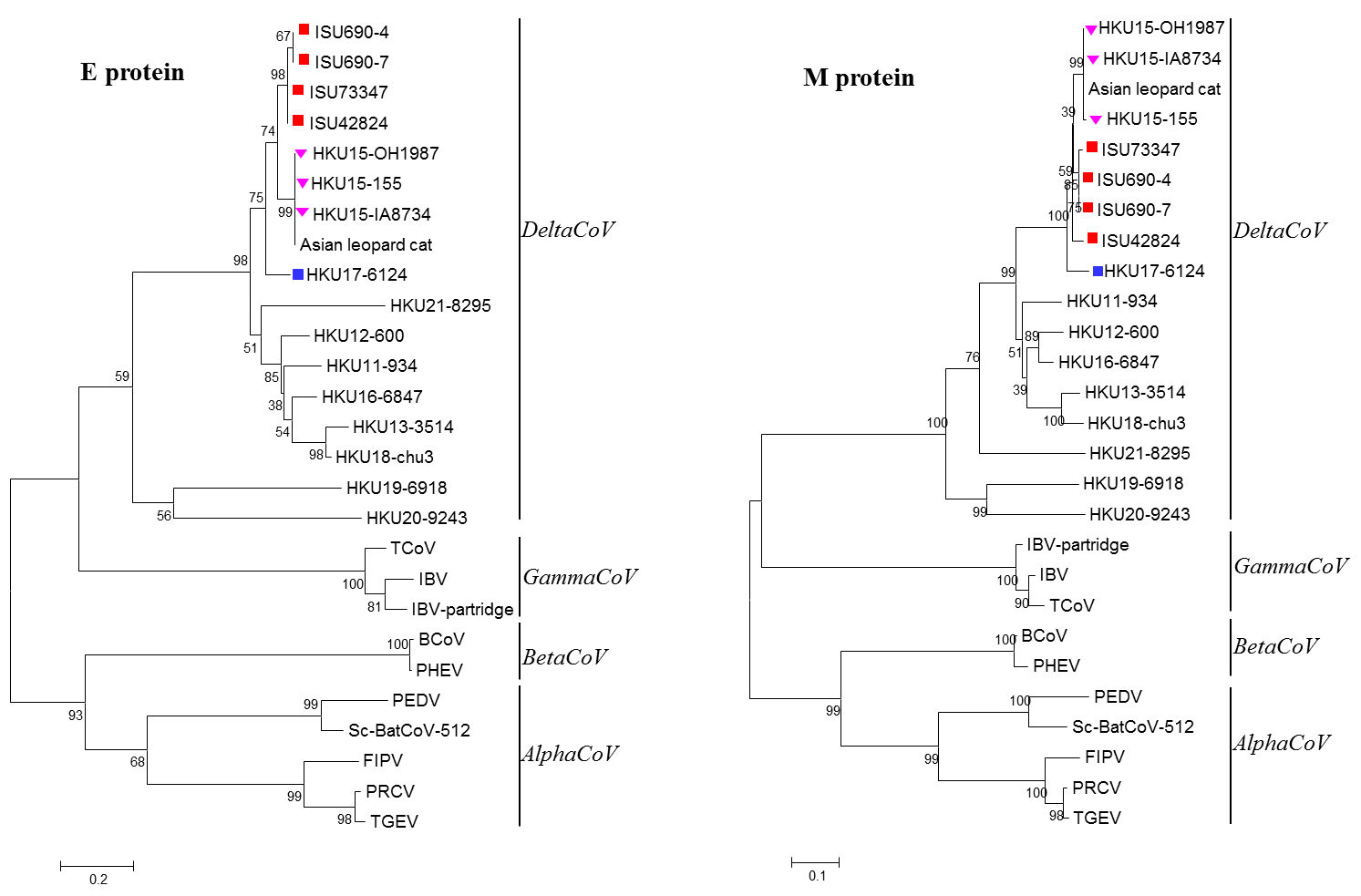


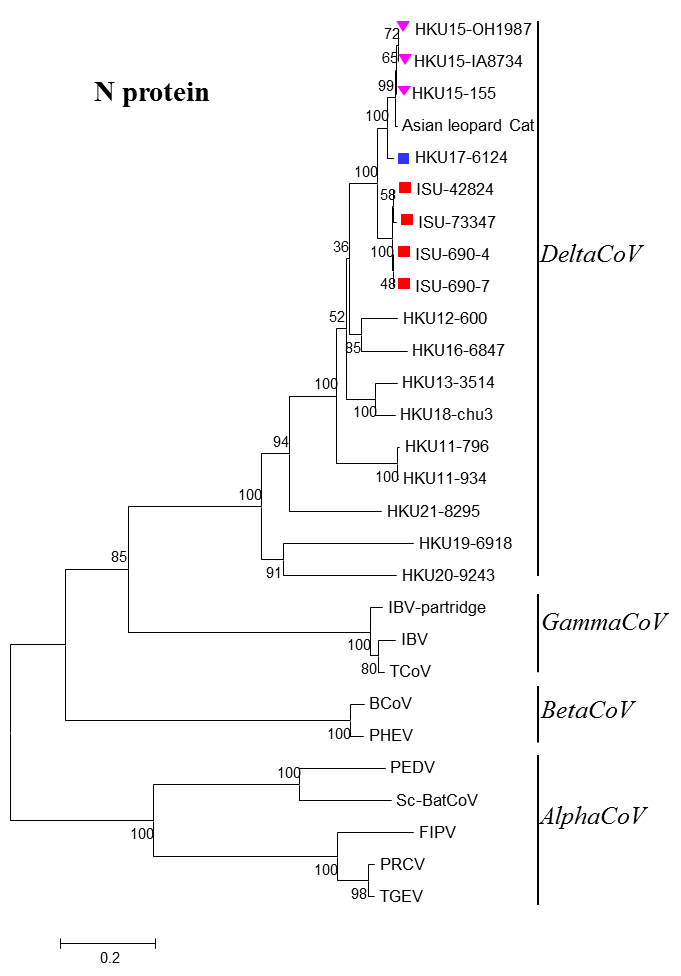


**Supplementary Figure S1**. Phylogenetic tree of amino acid sequences of E, M, and N proteins. The novel sparrow deltacoronavirus strains identified in this study were indicated with red squares, HKU17-6124 was marked with a blue square, and three HKU15 strains marked with a pink triangle. The dendrogram was constructed by using the neighbor-joining method in MEGA version 7.0. Bootstrap resampling (1,000 replications) was used, and bootstrap values are indicated for each node. Reference sequences were obtained from GenBank (PEDV, porcine epidemic diarrhea virus, NC_003436; Sc-BatCoV-512, *Scotophilus* bat coronavirus 512, NC_009657; TGEV, transmissible gastroenteritis virus, AJ271965; FIPV, feline infectious peritonitis virus, NC_002306; PRCV, porcine respiratory coronavirus, DQ811787; PHEV, porcine hemagglutinating encephalomyelitis virus, DQ011855; BCoV, bovine coronavirus, NC_003045; IBV, infectious bronchitis virus, NC_001451; IBV-partridge, partridge coronavirus, AY646283; TCoV, turkey coronavirus, NC_010800; bulbul CoV strain HKU11-796, FJ376620; bulbul CoV strain HKU11-934, FJ376619; thrush CoV strain HKU12-600, NC_011549; munia CoV strain HKU13-3514, NC_011550; PDCoV strain HKU15-OH1987, KJ462462; PDCoV strain HKU15-IA8734, KJ567050; PDCoV strain HKU15-155, JQ065043; white-eye CoV strain HKU16-6847, JQ065044; sparrow CoV strain HKU17-6124, NC_016992; magpie robin CoV strain HKU18-chu3, NC_016993; night heron CoV strain HKU19-6918, NC_016994; wigeon CoV strain HKU20-9243, JQ065048; common moorhen CoV strain HKU21-8295, NC_016996). Scale bar represents 0.2, 0.1, 0.2 amino acid substitutions per site for E, M, and N trees, respectively.


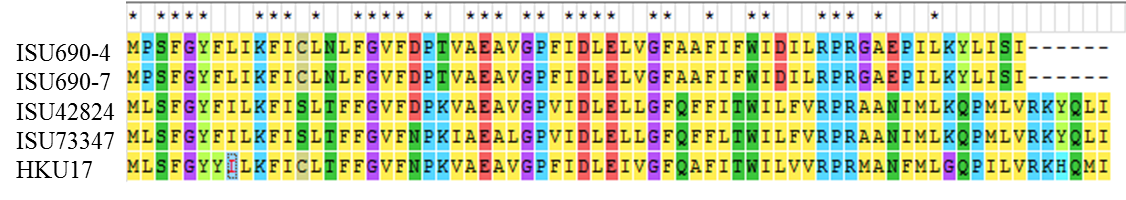


**Supplementary Figure S2**. Sequence alignment of NS7c amino acid sequences of four SpDCoV and HKU17-6124


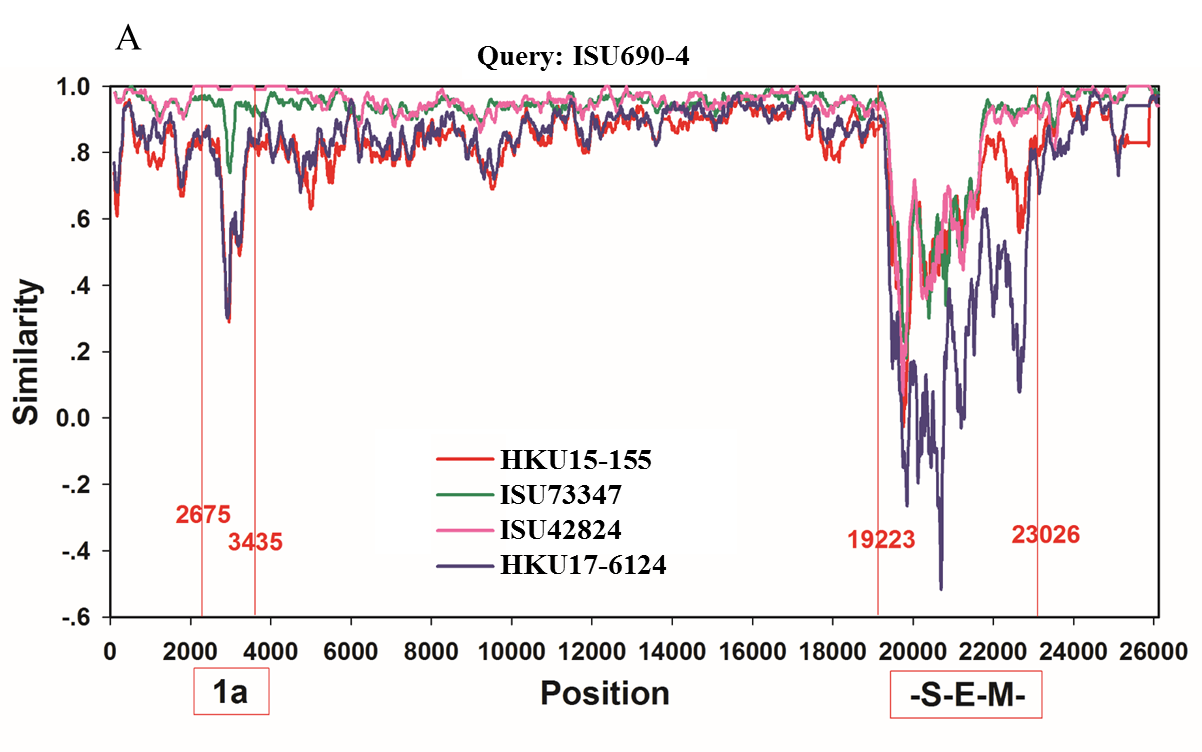


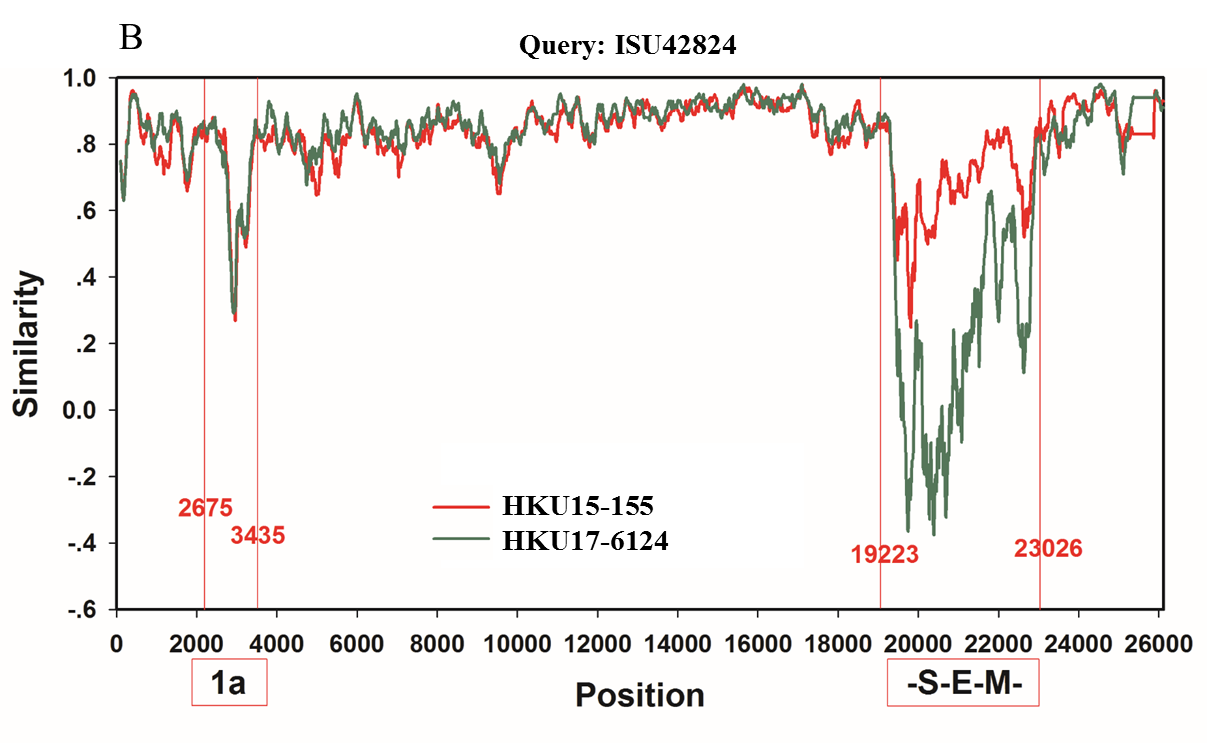


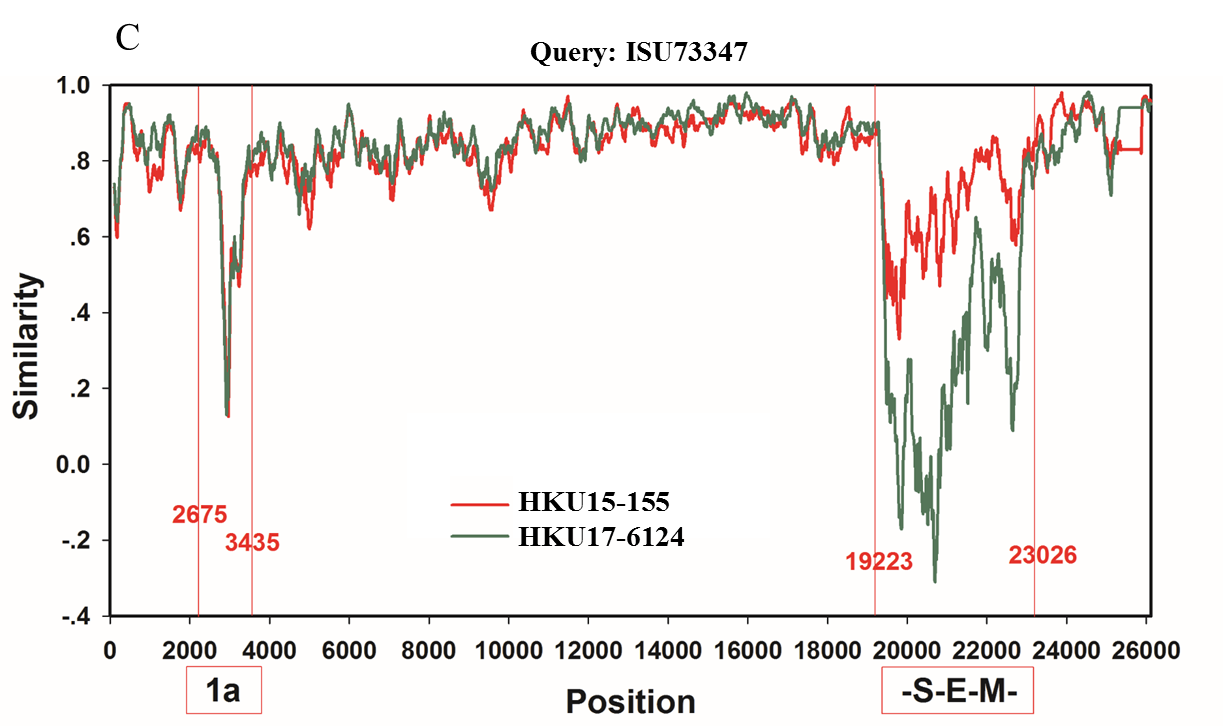


**Supplementary Figure S3.** Recombination analysis of complete genomes of ISU690-4, ISU42824, ISU73347, PDCoV HKU15-155, and sparrow CoV HKU17-6124, with ISU690-4 as query (A); complete genomes of ISU42824, PDCoV HKU15-155, and sparrow CoV HKU17-6124, with ISU42824 as query (B); complete genomes of ISU73347, PDCoV HKU15-155, and sparrow CoV HKU17-6124, with ISU73347 as query (C). Recombination analysis was performed with SimPlot V3.5.1. PDCoV and sparrow CoV are highly variable from SpDCoVs in 2 regions. The most prominent variable region is located in S-E-M genes, and another region is located in 1ab gene (~2675nt to 3435nt).


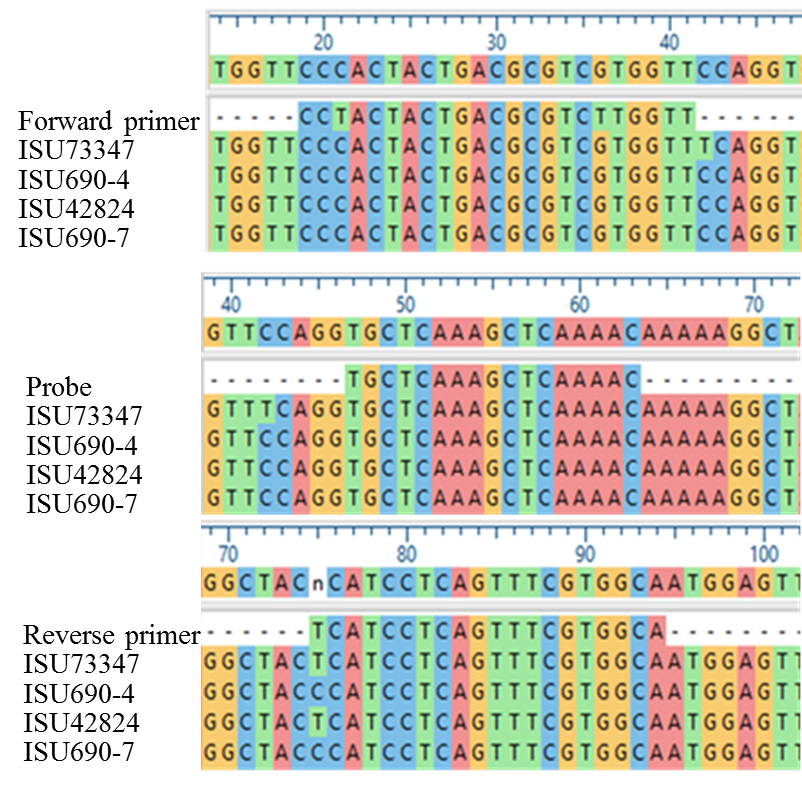


**Supplementary Figure S4**. Alignment of sequences of primers and probe of PDCoV real time RT-PCR with 4 SpDCoV strains.
